# P-1241. Pharmacokinetic Comparison of Augmentin ES-600 and a Reduced Concentration Clavulanate Formulation in Young Children; Modeling of amoxicillin/clavulanic acid in vitro effects on bacterial growth and killing and in vivo exposure evaluation

**DOI:** 10.1093/ofid/ofaf695.1433

**Published:** 2026-01-11

**Authors:** Keith Dewedoff, Alejandro Hoberman, Tomoyuki Mizuno, Alexander "Sander" vinks

**Affiliations:** Kaizen Biosciences, Philadelphia, New Jersey; University of Pittsburgh School of Medicine, Pittsburgh, Pennsylvania; Cincinnati Children's Hospital Medical Center, Cincinnati, OH; Cincinnati Children’s Hospital Medical Center, Cincinnati, Ohio

## Abstract

**Background:**

Amoxicillin/clavulanic acid is currently the most effective antimicrobial for the treatment of children with acute otitis media and recurrent otitis media. While the FDA-approved pediatric formulation, Augmentin ES-600^®^ (amoxicillin/clavulanic acid at 45/3.2 mg/kg), is highly effective, it is also known to cause problematic diarrhea, delaying children and their parents from resuming daily activities. A reduced clavulanic acid dose may mitigate these side effects without compromising therapeutic efficacy

**Methods:**

. In our study, we employed a model-informed approach to estimate amoxicillin/clavulanic acid exposure in middle ear fluid (MEF) and evaluated the suitability of a lower clavulanic acid dose (1.425 mg/kg) based on *in vitro* exposure-response (E-R) data. Using established pediatric PK models for oral amoxicillin/clavulanic acid, we simulated drug exposures in MEF for a virtual pediatric population (ages 3-24 months) derived from the NHANES database. *In vitro* bacterial growth and killing data against five isolates of *Haemophilus influenzae* were used to determine effective AUC/MIC metrics, which were then compared with the simulated exposures in MEF.

**Results:**

Our results suggest that the simulated mean AUC_0-24_ exceeds the *in vitro* AUC_0-24_/MIC required to achieve net bacterial stasis and 1-log 10 CFU/mL reduction against an isolate with an MIC of 0.5 mg/L in children 3-24 months of age. These findings indicate that a reduced clavulanic acid dose may provide effective therapeutic exposure in MEF while potentially mitigating adverse events.

**Conclusion:**

The significantly elevated clavulanic acid exposure associated with Augmentin ES-600 exceeds adult recommendations and raises questions about currently marketed amoxicillin: clavulanate ratios, suggesting the need to reassess dosing strategies to optimize efficacy and safety in young children

**Disclosures:**

Keith Dewedoff, n/a, Kaizen Biosciences: Board Member|Kaizen Biosciences: Ownership Interest|Kaizen Biosciences: Stocks/Bonds (Private Company) Alejandro Hoberman, MD, Kaizen Biosciences: Advisor/Consultant|Kaizen Biosciences: Stocks/Bonds (Private Company)

